# Automatic Imitation in Rhythmical Actions: Kinematic Fidelity and the Effects of Compatibility, Delay, and Visual Monitoring

**DOI:** 10.1371/journal.pone.0046728

**Published:** 2012-10-05

**Authors:** Daniel L. Eaves, Martine Turgeon, Stefan Vogt

**Affiliations:** 1 Sport and Exercise Science Section, Teesside University, Middlesbrough, United Kingdom; 2 Centre for Research in Human Development and Learning, Department of Psychology, Lancaster University, Lancaster, United Kingdom; Centre national de la recherche scientifique, France

## Abstract

We demonstrate that observation of everyday rhythmical actions biases subsequent motor execution of the same and of different actions, using a paradigm where the observed actions were irrelevant for action execution. The cycle time of the distractor actions was subtly manipulated across trials, and the cycle time of motor responses served as the main dependent measure. Although distractor frequencies reliably biased response cycle times, this imitation bias was only a small fraction of the modulations in distractor speed, as well as of the modulations produced when participants intentionally imitated the observed rhythms. Importantly, this bias was not only present for compatible actions, but was also found, though numerically reduced, when distractor and executed actions were different (e.g., tooth brushing vs. window wiping), or when the dominant plane of movement was different (horizontal vs. vertical). In addition, these effects were equally pronounced for execution at 0, 4, and 8 s after action observation, a finding that contrasts with the more short-lived effects reported in earlier studies. The imitation bias was also unaffected when vision of the hand was occluded during execution, indicating that this effect most likely resulted from visuomotor interactions during distractor observation, rather than from visual monitoring and guidance during execution. Finally, when the distractor was incompatible in both dimensions (action type and plane) the imitation bias was not reduced further, in an additive way, relative to the single-incompatible conditions. This points to a mechanism whereby the observed action’s impact on motor processing is generally reduced whenever this is not useful for motor planning. We interpret these findings in the framework of biased competition, where intended and distractor actions can be represented as competing and quasi-encapsulated sensorimotor streams.

## Introduction

In everyday life, we often mimic the postures and gestures of the people we interact with, typically without knowing or meaning to do so. This behavioural mimicry is also known to impact upon cooperative social attitudes [1]. There is now a substantial body of research into the neurocognitive mechanisms of such imitative phenomena, which essentially indicates that observing another person’s actions primes similar actions in the observer (visuomotor priming, for a review see [2]). More recently, this phenomenon has been termed ‘automatic imitation’ [3]: a type of stimulus-response compatibility (SRC) effect, wherein pre-instructed actions are initiated faster when observing a congruent compared to an incongruent distractor action (e.g., [4]–[7]). Using this approach, researchers have tackled a number of important issues, such as the distinctness of automatic imitation from spatial compatibility effects, and different criteria of automaticity [3]. A further proposal is that automatic imitation might represent a laboratory model of the behavioural mimicry found in more naturalistic social settings [3]. Unlike behavioural mimicry research, however, the reaction time (RT) methodology typically used in studies on automatic imitation does not convey information about the degree of similarity between an observed action and the related response. We address this under-researched issue by studying a core aspect of imitative alignment, namely *rhythmicity*. While rhythmic alignment is also likely to represent one of the main behavioural manifestations of ‘motor resonance mechanisms’ in the brain [2], [8], in the present study we focus on observable behaviour at first.

Imitation is a multi-level phenomenon. On the one hand, research has demonstrated that high-level behavioural goals, and not necessarily the detailed means to achieve them, are central to imitation. This is not only the case in imitation learning [9]–[12], but also in instantaneous imitative behaviour (e.g., [13]). On the other hand, behavioural studies also indicate that certain fine kinematic details of an observed action, often irrelevant for goal achievement, are automatically encoded and bias motor execution (e.g., [14], [15]). The present study focuses on this latter aspect. In addition, we assessed the extent to which such low-level imitation depends on goal congruency, as well as on the spatial correspondence between observed and executed actions.

Perhaps some of the clearest examples of low-level sensorimotor couplings come from research adopting a dynamical systems approach to rhythmical actions. In *online* synchronisation paradigms (i.e., simultaneous action observation and execution), rhythmical actions exhibit a spontaneous tendency to phase-entrain, both for within-person [16] and for between-person coordination [17], even when unintended [18]. Oullier et al. [19] further showed that, following phase-entrainment, dyads preserved their new shared frequency (without instruction to do so) when vision was occluded over a 1 min observation period; an effect those authors termed ‘social memory’.

While dynamical systems research has focused on online synchronisation, a separate branch of behavioural research has demonstrated an imitation bias in *offline* imitation paradigms (i.e., motor execution subsequent to action observation). To date, the following kinematic parameters have been studied: finger-tapping frequency [20], grip force [21]–[22], pointing velocity [23]–[24] and accuracy [25], object-transport velocity [26], reach-grasp trajectory [14], [27], as well as its velocity and grip aperture [15], [28]–[31]. These studies have shown that the imitation of kinematic details is sensitive to the observer’s *a priori* goals and to the action context [15], to the presence of spatial targets in the demonstrated action [24], to the proximity of the observed action to the observer’s peripersonal space [14], to changes in observed biological over non-biological velocity profiles [23], and to observed movement errors [25]. Furthermore, the imitation bias is not effector-dependent [28], and it can also influence the subsequent estimation of object properties [31]. However, in most of these studies the *kinematic fidelity* (i.e., the degree of similarity) between observed and executed actions was not quantified. In contrast, this was the principal aim of the present offline imitation study. Next we expand on the two main objectives for our study, and highlight a few key findings from the research listed above.

Our first aim was to quantify automatic imitation for rhythmical actions as the kinematic fidelity (a) between observed and executed actions, and (b) relative to performance in intentional imitation of the observed rhythm. We used an extended SRC paradigm where participants performed one out of a set of eight everyday rhythmical actions (‘imperative action’) after observation of a short, action-irrelevant rhythmical distractor movie of the same or a different action. Across trials, the cycle times of the distractor actions were subtly manipulated (slow or fast). This allowed us to quantify kinematic fidelity as the ratio between executed and observed cycle times.

Two recent studies provide data related to our first aim. In Bisio et al.’s [23] study, participants executed discrete pointing actions to one of two targets after observing a model perform the same action. Covert manipulation of the model’s reach velocity biased response velocities, relative to a condition without action observation. The magnitude of this imitation bias was roughly half of that observed for intentional imitation. Bove et al.’s [20] participants passively observed either a slow (1 Hz) or a fast (3 Hz) rhythmical finger-tapping action for 10 min. Immediately thereafter, finger-tapping frequencies in both the slow and fast groups differed from a no-action-observation group. Across action observation groups, this bias was approximately 65% of that for intentional imitation. However, the data from these two studies might not provide adequate indices for the kinematic fidelity of automatic imitation, since their distractor actions were always the same as the to-be-performed action (albeit with different speed parameters). Consequently, participants could have reinterpreted these ‘task-irrelevant’ distractors as a task-relevant guide for action. This strategic coupling of motor preparation to the available visual input could then also have included the kinematic properties of the distractor. As a result, the effects reported in these two studies will most likely not reflect the kinematic fidelity of *automatic* imitation, given that the defining characteristic of the effect is that it occurs independently of intention [3]. We addressed this issue by assessing the imitation bias both for distractor actions that resembled the imperative action (as in the above studies), and for distractor actions that differed from the imperative actions along two dimensions, as discussed next.

Our second aim was to study the imitation bias for two dimensions of compatibility, namely action type (or action goal), and plane of movement (horizontal or vertical). At present we are unaware of any studies that have directly manipulated action type compatibility in this context. It is thus unclear to what extent incompatible distractor actions might affect the imitation bias relative to compatible actions. To this end, we displayed Different Actions performed in the Same Plane (DA/SP) of movement as the imperative stimuli, in addition to a fully compatible Same Action/Same Plane (SA/SP) condition. A second factor that might modulate the imitation bias is spatial compatibility. For example, the kinematics of rhythmical arm movements have been shown to be biased by the simultaneous presentation of an orthogonal rhythmical distractor [32]–[35]. In addition, Hove et al. [36] showed a tighter coupling between finger-tapping and a visual rhythm when these were spatially compatible (e.g., simultaneously downwards), rather than in orthogonal planes. While these studies demonstrate spatial compatibility effects in online synchronisation, we studied these effects in our offline paradigm, using a Same Action/Different Plane (SA/DP) condition. According to the above considerations, one would expect that both compatibility dimensions, action type and plane, affect the strength of the imitation bias to some extent, where genuine automatic imitation is assessed only via the incompatible conditions. Furthermore, we were interested in whether the detrimental effects of action and plane compatibility, if found, would combine in an additive manner when action type and plane were both incompatible in a fourth, Different Action/Different Plane (DA/DP) condition.

Note that we have just described the possible effects of incompatible action types and of incompatible planes separately from our earlier argument, namely that in trials where instructed and distractor actions are compatible, participants might simply copy the distractor action. However, these two explanations most likely coincide, at least in the present paradigm: the very reason why incompatible action types and planes give rise to smaller effects than compatible distractors might be that the former are not suitable for concurrent action planning. This point will be developed further in the Discussion.

In addition to the two main research aims described above, we manipulated two further variables: the delay between distractor presentation and execution, and the opportunity for visual monitoring of one’s own hand during execution (the latter as a between-subjects factor). For brevity, the rationale for these additional manipulations is explained, together with the related results, in the Discussion. In summary then, we addressed the following four research questions in the present study:

1. What is the kinematic fidelity of the automatic imitation of rhythm, assessed
  - as the gain between cycle time ratios in observed and executed actions, and
  - relative to motor performance in intentional imitation of the observed rhythms?
2. Are automatic imitation effects reduced when imperative and distractor actions differ regarding (a) action type, (b) plane, and (c) both compatibility dimensions?
3. Do automatic imitation effects persist over a 4 s and 8 s delay between action observation and execution?
4. Does automatic imitation rely on visual monitoring of the hand during execution?

## Methods

We assessed automatic imitation in Experiment 1, which consisted of two sessions run on consecutive days (see Method, Sections 1 to 4). Intentional imitation was assessed in Experiment 2, which consisted of one session that was run 7 to 14 days after Experiment 1 (see Method, Section 5).

### 1. Participants

Twenty participants (13 male, mean age 27.2 yrs; SD = 5.9 yrs) volunteered for the study. All had normal (*n* = 13) or corrected-to-normal vision. Participants were naïve to the study’s purpose, right-hand dominant (Edinburgh Handedness Inventory: *M* = 87; [37]), and without physical injuries. They were randomly allocated to one of two vision conditions, either full vision throughout all procedures, or were asked to close their eyes during motor execution. Written informed consent was obtained prior to participation, and ethical approval had been granted by Teesside University and Lancaster University.

### 2. Task and Design

**Figure 1:**
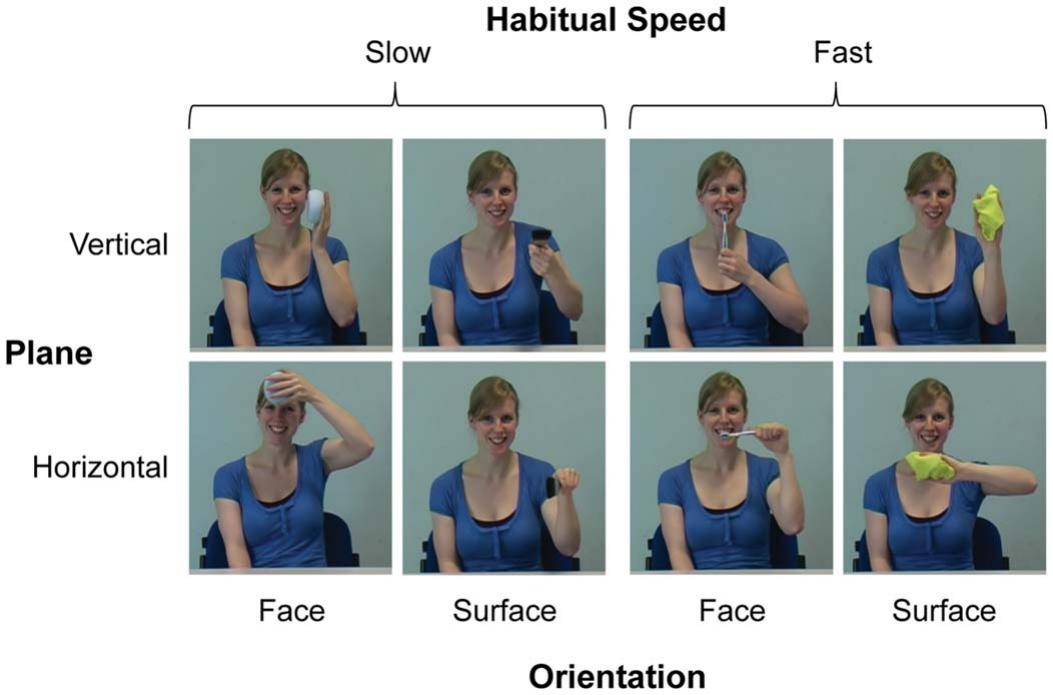
Imperative action stimuli with the factors plane, habitual speed, and orientation.

In each experimental trial, participants watched a picture of a to-be-pantomimed everyday rhythmical action (imperative stimulus, see Figure 1 and Method, Section 3), followed by a short, action-irrelevant distractor movie of the same or a different action. They then executed the imperative pantomime action. We studied actions that are typically performed relatively slow (‘habitually slow actions’) as well as habitually fast actions. Within each habitual speed category, slow and fast versions of each distractor action were used.

Experiment 1 consisted of six blocks of 32 trials, with three blocks run in each of the two sessions. A six-factorial mixed design was used. The availability of vision during execution was manipulated between subjects (vision vs. no-vision). The delay between action observation and execution was manipulated across the three blocks run in each session (0, 4, or 8 s), in a counterbalanced order across participants. The other four factors were manipulated within each block of trials: habitual action speed (slow or fast), distractor speed (slow or fast), action type compatibility (same or different action: SA or DA), and plane compatibility (same or different plane: SP or DP) between imperative and distractor actions. The two compatibility factors thus yielded four levels of compatibility: SA/SP, SA/DP, DA/SP, and DA/DP.

Note that the two factors of action type compatibility and plane compatibility were derived from pooling the data from their four constituent factors, namely: imperative action type (face- or surface-oriented, see Figure 1 and Method, Section 3), imperative action plane (horizontal or vertical), distractor action type, and distractor action plane. The full combination of these four factors with habitual action speed and distractor speed resulted in 64 trials for each of the three delay conditions, half of which were presented in a quasi-random order within each block of session one, and the other half in session two. As a result of the pooling, each cell of the effective six-factorial design consisted of an average across four trials.

### 3. Stimuli and Apparatus

A conventional digital video camera (Panasonic NV-MX500B) was used to create the imperative picture and distractor movie stimuli. Figure 1 shows the eight imperative stimuli: The two habitually slow actions were face washing and paint brushing, and the two habitually fast actions were tooth brushing and window wiping. Within each habitual speed category, one face-orientated and one surface-orientated action was used, each of which could be performed in either the vertical or in the horizontal plane. Given the relatively complex design, and given that we were only interested in the compatibility between imperative and distractor actions regarding action type and plane, rather than in the separate effects for each individual action, we pooled the data across imperative action types and planes (as described above). The model performed all actions with the left hand to provide mirror images of the participants’ subsequent actions, who always used their right hand. This arrangement provided spatial compatibility between displayed and performed actions, which has been shown to facilitate imitation relative to an anatomically matched but spatially incompatible arrangement (e.g., [11], [38]).

Sixteen distractor movies were used in the main experiment, one slow and one fast version of each of the eight imperative actions. During filming, the model’s performance had been paced by a metronome to achieve the exact distractor speeds shown in Table 1, whereas throughout the experiment all stimuli were displayed without sound. Importantly, imperative stimuli were always paired with a distractor stimulus from within the same habitual speed category. We used two habitual speeds for two reasons: First, we wanted to assess the imitation bias of the distractor movies on motor execution across a range of cycle times and not just for one speed. Second, the fact that participants executed, in quasi-random order, rhythmical actions with two substantially different habitual speeds served to divert their attention away from the more subtle manipulation of the distractor speeds. Finally, note that each imperative action was displayed with the relevant object (sponge, paintbrush, toothbrush, or cloth), which enabled quick discrimination between the actions, whereas participants performed pantomimed actions (without objects). The latter was done to avoid participants having to select the relevant object in the beginning of each trial. The distractor movies showed pantomimed actions to allow participants to better distinguish between imperative and distractor stimuli, and to potentially strengthen the impact of the distractor stimuli on the subsequent pantomimed execution.

**Table 1:** Distractor stimuli specifications.

Parameters	Habitually slow actions		Habitually fast actions	
Distractor speed	Slow	Fast	Slow	Fast
Beats per min	60	90	120	180
Cycle times (ms)	1000	667	500	333
Total cycles in 4 s	4	6	8	12
Slow:fast ratio (%)	150		150	

Participants sat at a wooden desk in a dimly-lit room facing a 17-in LCD computer monitor (Apple Studio Display) positioned approx. 80 cm away from their head. All stimuli were displayed against a light grey background via PsyScript 2.3 software (http://www.psych.lancs.ac.uk/software/psyScript.html) running on a Power Macintosh G4 computer fitted with a digital I/O board. The start location for the participants’ right index finger and thumb was on an electro-conductive plate mounted on top of a 23 cm-tall wooden post, 20 cm ahead of them on the desk. A magnetic motion sensor was fitted to the distal end of the second metacarpal bone of the right hand. Participants’ kinematic data were sampled at 103 Hz in 3-D space for 4 s periods using a Minibird Magnetic Tracking System (Ascension Technology Corp.), and were stored on a separate PC. At the end of each trial, kinematic data plots were displayed on a second monitor, unseen by participants.

### 4. Procedure

#### Familiarisation

In *Phase 1* of the familiarisation period, participants learned to pantomime each action from a set of eight familiarisation movies (eight actions with two attempts each). These movies were identical to the movies for the main experiment, except that the cycle times were mid-way between the distractor speeds shown in Table 1, that is, 75 bpm for the habitually slow actions, and 150 bpm for the habitually fast actions. Participants were given verbal feedback about their movement based on the kinematic plots visible to the experimenter. This ensured that their movement amplitude and cycle time aligned closely with the medium-paced stimuli. In *Phase 2*, participants saw a picture of each action while simultaneously pantomiming the same action for 4 s (16 trials). In *Phase 3*, they experienced the structure of trials in the main experiment, including the four compatibility conditions, but using the medium-paced distractors (16 trials). In Phases 2 and 3, verbal feedback was only given if movements occasionally drifted away from the criterion amplitude (10 cm for all actions) or cycle times. Short versions of this familiarisation procedure were run on each new day of testing.

#### Main experiment

**Figure 2:**
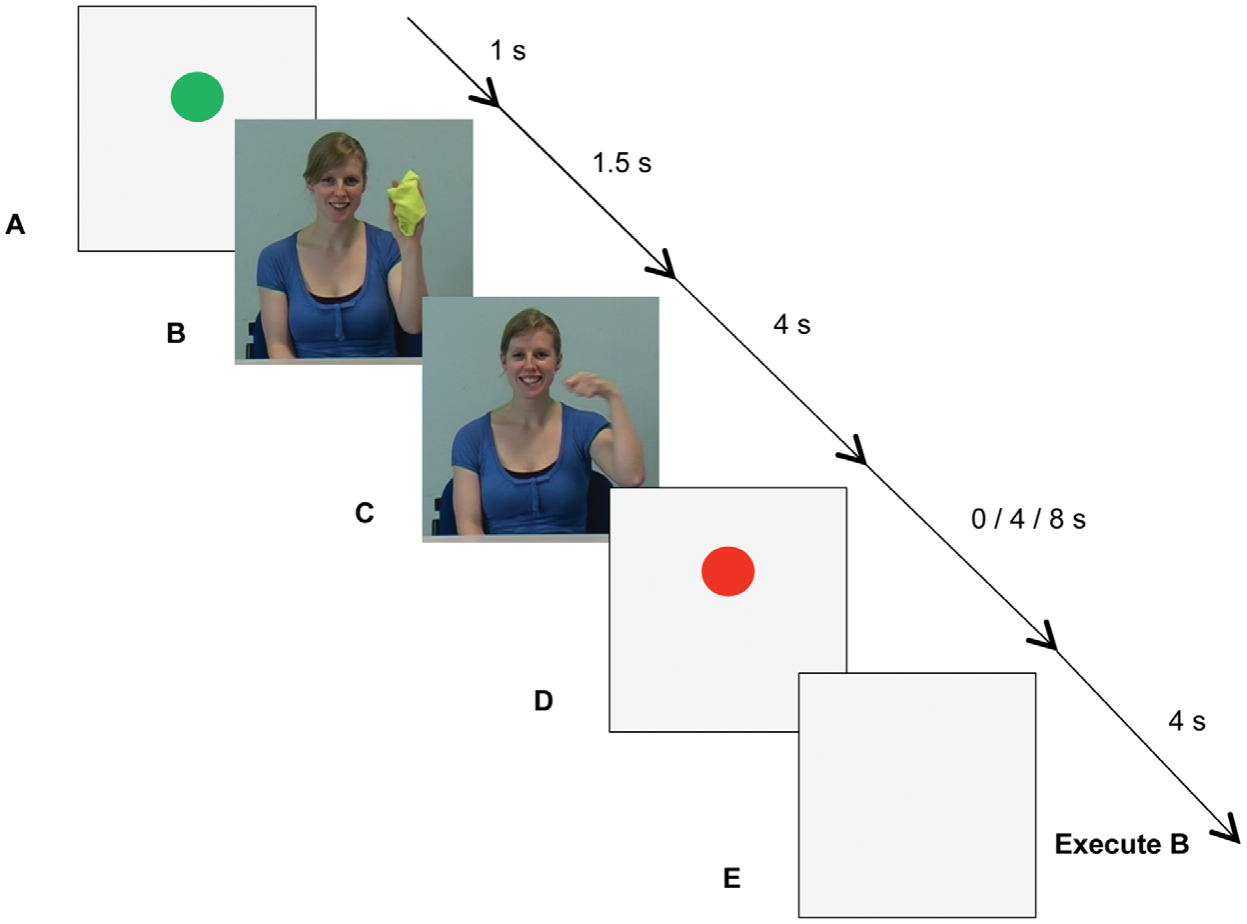
Sequence of events in the automatic imitation experiment. (A) A green circle appeared when participants placed their fingers in the start location. (B) Then a picture of the to-be-pantomimed (imperative) action was shown, followed by (C) a distractor movie of the model pantomiming either the same or a different action. (D) In blocks with delayed execution, a red circle was shown for either 4 or 8 s. (E) Execution of the imperative action was cued by display of a neutral, light-grey background, which appeared at the offset of either the distractor movie or the red circle.

When participants placed their fingers in the start location, a green circle appeared on the monitor for 1 s to mark the beginning of a trial (Event A in Figure 2). (B) Then a picture of the to-be-pantomimed (imperative) action was shown for 1.5 s, followed by (C) a distractor movie of the same model pantomiming either the same or a different action. During movie presentation, participants fixated on the model’s left eye to minimise any visual coupling to the model’s rhythmical arm movements [39]. (D) In blocks with delayed execution, a red circle was shown for either 4 or 8 s. (E) Finally, the imperative action was performed while movement kinematics were tracked in 3-D. The end of the 4 s recording interval was indicated by a computer-generated auditory signal, after which participants could verbally report distractor characteristics (see below) before moving their hand back to the start location.

The core manipulation across trials was that of distractor speed, with a ratio of slow:fast movements of 150% (see Table 1). Participants were not informed of the distractor speed changes, and this manipulation was further concealed by the more prominent differences between the two habitual speeds across trials. To focus their attention on the distractor movie, participants were asked to verbally recall the distractor properties (action type and plane) after motor execution on approx. 10% of trials. Experiment 1 was distributed over two sessions to reduce the possibility of physical fatigue from prolonged testing. Each session consisted of three blocks of 32 trials (see Method, Section 2), and each block was preceded by a single warm-up trial and followed by a rest period of 5 min.

### 5. Experiment 2: Intentional imitation

Design and procedures of the intentional imitation experiment were essentially the same as those for the automatic imitation experiment described above, with the following differences: First, participants were asked to execute the imperative actions while imitating the cycle times of the distractor movies as precisely as possible. Second, we only assessed the 0 s delay condition, for which intentional imitation should be optimal. Experiment 2 thus involved only two blocks of 32 trials. As before, participants fixated on the model’s left-eye during distractor observation, but they did not verbally recall the distractor actions between trials.

### 6. Data Analysis

Mean cycle times (ms) were calculated between peak maximum kinematic positions using a customised signal processing tool within Matlab (Mathworks, Inc., Natick, MA). For both horizontal and vertical actions, the first data point taken was the peak maximum of the second movement cycle. The first cycle was not used as this additionally reflected the spatial positioning of the hand before a stable workspace was reached. Mean cycle time was calculated across all peak positions available within a 2000 ms time window across all speed conditions. This involved either two or three cycles for habitually slow actions and four or five cycles for habitually fast actions. All trials with erroneous responses (incorrect or no action) were discarded (*n* = 41).

The two main dependent measures were the mean cycle time (ms) and the ratio (%) between slow and fast distractor trials. While the absolute difference between distractor cycle times was greater in the habitually slow actions (667 vs. 1000 ms) compared to the habitually fast actions (333 vs. 500 ms), the ratio of slow:fast distractor speeds was the same for each habitual speed (150%). For economy of exposition, we therefore restricted the analysis of the mean cycle time data to three factors of interest, and analysed the additional effects of delay and of the compatibility manipulations only for the cycle time ratios. Accordingly, the mean cycle times (ms) were analysed via a three-factorial, mixed-measures ANOVA with the two within-subjects factors distractor speed (only available for this measure) and habitual speed, and with the availability of vision as the between-subjects factor. The cycle time ratios (%) were subjected to a five-factorial mixed-measures ANOVA, with habitual speed, action type compatibility, plane compatibility, and delay as the within-subjects factors, and the availability of vision as the between-subjects factor. Subsequently, both dependent measures were analysed for the intentional imitation experiment. Finally, we contrasted automatic and intentional imitation using a five-factorial ANOVA on the cycle time ratios only, again for economy of exposition. All analyses were conducted using SPSS Statistics 19 (IBM). Where appropriate, these were adjusted for any violation of the homogeneity of variance assumption using the Greenhouse–Geisser correction. Alpha levels were set to 0.05, and effect sizes were calculated as partial eta squared values (*η_p_^2^*).

Reaction time data were also recorded on each trial as the time taken from response-cue onset to movement onset. These data were analysed, but the effects obtained were either trivial or unpredicted, and they did not directly address the aims of this paper. Therefore, these results are not reported here. However, RTs were used to identify trials with anticipatory (<200 ms; *n* = 14) or omission errors (>1300 ms; *n* = 68), which were discarded from all analyses. In total, 2.4% of all trials recorded were removed from the analyses.

## Results

### Experiment 1: Automatic Imitation

**Figure 3:**
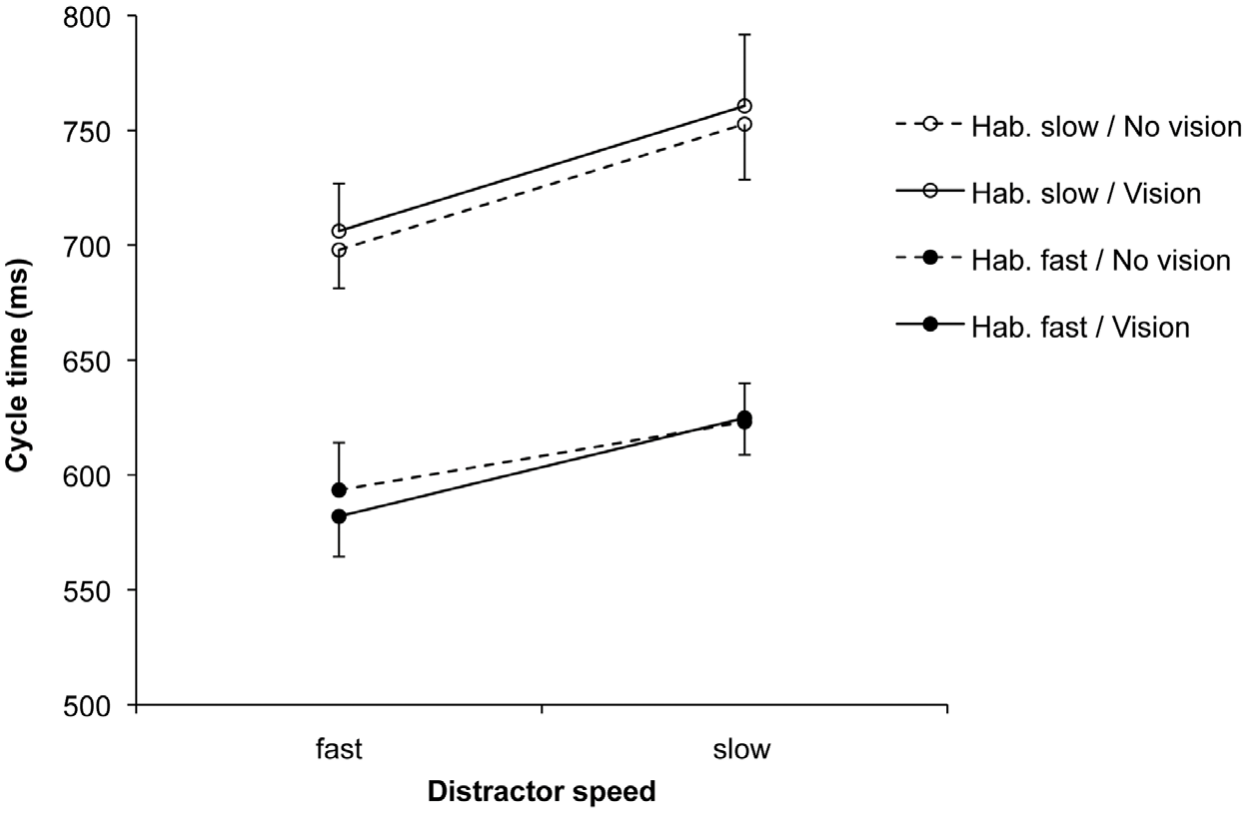
Automatic imitation experiment: Cycle times (ms). Mean cycle times for the factors habitual speed, distractor speed, and the availability of vision of the hand. The error bars show the standard error of the mean.

The three-factorial ANOVA on the cycle time (ms) data yielded a significant main effect of distractor speed, *F*(1, 18) = 47.07, *p*<0.001, *η_p_^2^* = 0.72. As predicted, response cycle times were shorter after seeing a fast, compared to a slow distractor (645 vs. 690 ms; see Figure 3). Trivially, the main effect of habitual speed was also significant, *F*(1, 18) = 68.57, *p*<0.001, *η_p_^2^* = 0.79. There was no significant main effect for the availability of vision, *F*(1, 18) = 0.01, *p>*0.05. The only significant interaction was that between distractor speed and habitual speed, *F*(1, 18) = 12.45, *p*<0.01, *η_p_^2^* = 0.41. This reflected the fact that, although the ratio of slow:fast distractor speeds was the same for each habitual speed (150%), the absolute difference between distractor cycle times was greater in habitually slow actions compared to habitually fast actions (see Method, Section 6).

**Figure 4:**
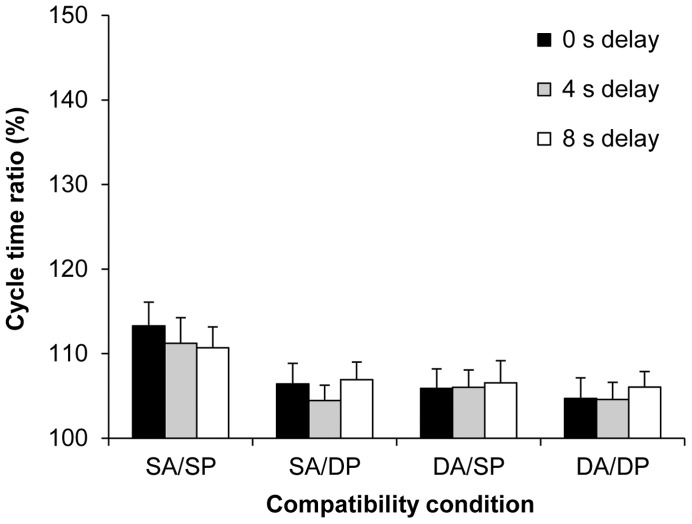
Automatic imitation experiment: Cycle time ratios (%). Mean cycle time ratios (with standard error of the mean) for the factors delay, action type compatibility, and plane compatibility (SA = Same Action; DA = Different Action; SP = Same Plane; DP = Different Plane). The cycle time ratio in the distractor actions was 150%.

The five-factorial ANOVA on the cycle time ratio (%) data yielded a significant main effect of action type compatibility, *F*(1, 18) = 22.78, *p*<0.001, *η_p_^2^* = 0.56, and of plane compatibility, *F*(1, 18) = 34.56, *p*<0.001, *η_p_^2^* = 0.66. In both cases, the cycle time ratio was closer to the display ratio (150%) for compatible than for incompatible trials (109 vs. 106%; 109 vs. 105%, respectively; see Figure 4). Unexpectedly, the main effect of delay was not significant, *F*(2, 36) = 1.12, *p>*0.05. Different to the ANOVA on the mean cycle time data (ms), the effect of habitual speed was not significant in the cycle time ratios, confirming that the imitation bias was similarly pronounced at both habitual speeds, when expressed as cycle time ratios. Again, the main effect for the availability of vision was not significant.

Only two significant interactions were found. First, the interaction between action type and plane compatibility was significant, *F*(1, 18) = 16.23, *p*<0.01, *η_p_^2^* = 0.47. Pairwise comparisons using Bonferroni corrections showed that responses in the fully compatible SA/SP condition were biased significantly more by the different distractor speeds than responses under each of the other three incompatible conditions (in all cases *p*<0.001). In contrast, responses did not differ across the three incompatible conditions (all *p*s ≥0.99). Importantly, running separate simple effect analyses on the cycle time (ms) data confirmed a significant main effect of distractor speed in each of the three incompatible conditions (all *p*s <0.001, all *η_p_^2^*s ≥0.60).

The second significant interaction was that between delay and habitual speed, *F*(2, 36) = 3.66, *p<*0.05, *η_p_^2^* = 0.17. Simple effect analyses revealed that while the main effect of delay was not significant for the habitually fast actions, *F*(2, 36) = 1.02, *p>*0.05, this effect was marginally significant for the habitually slow actions, *F*(2, 29) = 3.39, *p = *0.057, *η_p_^2^* = 0.16. More detailed simple effect analyses showed that the main effect of delay was only significant for habitually slow actions when action type was compatible, *F*(2, 36) = 5.41, p<0.01, *η_p_^2^* = 0.23. Pairwise comparisons showed that the slow:fast ratios were more pronounced in the 0 s delay condition than in the 4 s and 8 s delay conditions (112%, 108%, and 109%, respectively, both *p*s <0.05). These results indicate a pocket of stronger distractor effects for immediate vs. delayed execution in a subset of the data (compatible action type, and habitually slow actions). Response ratios were not significantly different across the two extended delay conditions.

### Experiment 2: Intentional Imitation

**Figure 5:**
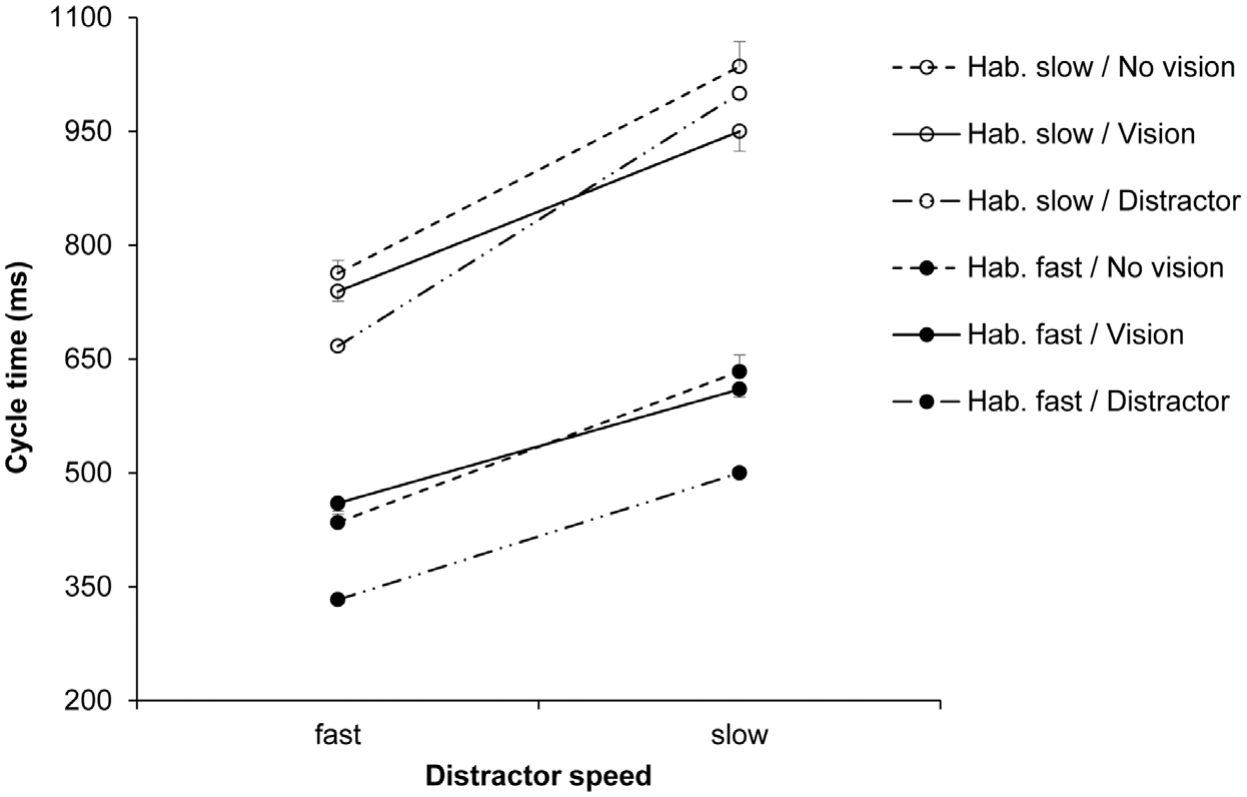
Intentional imitation experiment: Cycle times (ms). Mean cycle times (with standard error of the mean) for the factors habitual speed, distractor speed, and the availability of vision of the hand, displayed relative to the actual distractor speeds.

The three-factorial ANOVA on the cycle time (ms) data yielded significant main effects of distractor speed, *F*(1, 18) = 408.33, *p*<0.001, *η_p_^2^* = 0.96, and of habitual speed, *F*(1, 18) = 892.15, *p*<0.001, *η_p_^2^* = 0.98 (see Figure 5). Note that in intentional imitation participants were instructed to imitate the ‘distractor’ cycle times. For reasons of consistency, we continue using the term ‘distractor’ for these analyses. As one might expect for intentional imitation, the interaction between distractor speed and habitual speed was also significant, *F*(1, 18) = 49.74, *p*<0.001, *η_p_^2^* = 0.73, which was in line with the stronger cycle time differences (in ms) for the habitually slow over fast actions in the distractor movies. Again, the main effect for the availability of vision was not significant, *F*(1, 18) = 2.07, *p*>0.05. However, the availability of vision interacted both with distractor speed, *F*(1, 18) = 7.13, *p*<0.05, *η_p_^2^* = 0.31, and with habitual speed, *F*(1, 18) = 6.06, *p*<0.05, *η_p_^2^* = 0.25, while the three-way interaction was not significant. Simple effect analyses regarding the first of these two interactions showed that the availability of vision did not modulate intentional imitation of the fast distractor speeds, *F*(1, 18) = 0.002, *p*>0.05. But for the slow distractor speeds, response cycle times tended to be shorter when vision was available compared to unavailable (780 ms vs. 835 ms, respectively; criterion = 750 ms), *F*(1, 18) = 3.79, *p* = 0.067, *η_p_^2^* = 0.17; particularly for the habitually slow actions (945 vs. 1035 ms; criterion = 1000 ms), *F*(1, 18) = 4.86, *p*<0.05, *η_p_^2^* = 0.34. Next, simple effect analyses for the interaction between vision and habitual speed revealed a similar pattern, namely that the availability of vision did not modulate the habitually fast actions, *F* (1, 18) = 0.004, *p*>0.05, but for habitually slow actions, full-vision tended to speed-up response cycle times over no-vision (845 ms vs. 899 ms; criterion = 833 ms), *F*(1, 18) = 3.77, *p* = 0.068, *η_p_^2^* = 0.17. In summary, full-vision generally increased response speeds in intentional imitation, but also decreased the difference between response cycle times executed after both slow and fast distractors (see also below).

The four-factorial ANOVA conducted on the ratio (%) data for intentional imitation used the same factors as the related ANOVA for automatic imitation, except that the delay factor was not included. This ANOVA yielded a significant main effect of habitual speed, *F*(1, 18) = 11.16, *p*<0.01, *η_p_^2^* = 0.38. The slow:fast cycle time ratio was larger for the habitually fast (140%) over slow actions (132%), while both undershot the to-be-imitated ‘distractor’ ratio of 150%. Thus, while the ms-data reflected the time differences in the displays in both automatic and intentional imitation, this difference did not affect cycle time ratios in automatic imitation, but it did increase the ratios in intentional imitation for the habitually fast over slow actions. Also, the availability of vision significantly modulated the cycle time ratios, *F*(1, 18) = 8.14, *p*<0.05, *η_p_^2^* = 0.31, with a more pronounced mean ratio found in the no-vision (141%) compared to the vision condition (131%). The main effect of action type compatibility was not significant, *F*(1, 18) = 0.01, *p*>0.05, as was the main effect of plane compatibility, *F*(1, 18) = 0.93, *p*>0.05. The only significant interaction was between habitual speed and plane compatibility, *F*(1, 18) = 4.44, *p*<0.05, *η_p_^2^* = 0.20. Pairwise comparisons showed that for habitually slow actions the slow:fast ratio was marginally more pronounced for trials with compatible compared to incompatible plane (131 vs. 133%; *p* = 0.055).

### Automatic vs. Intentional Imitation

The five-factorial ANOVA performed on the ratio (%) data used the same factors as the four-factorial ANOVA for intentional imitation, except with the inclusion of a fifth factor ‘automatic vs. intentional imitation’. This analysis yielded a number of significant results that replicated our previous findings. As can expected from our separate analyses of the ratio scores for automatic and intentional imitation, significant main effects were found for action type, *F*(1, 18) = 8.12, *p* = 0.01, *η_p_^2^* = 0.31, and plane compatibility, *F*(1, 18) = 12.11, *p*<0.01, *η_p_^2^* = 0.40. Furthermore, two interaction results confirmed the already reported differences between automatic and intentional imitation: The significant interaction between intention and visual monitoring, *F*(1, 18) = 19.72, *p*<0.001, *η_p_^2^* = 0.52, confirmed that visual monitoring only affected cycle time ratios in intentional imitation, and the significant interaction between intention and habitual speed, *F*(1, 18) = 27.47, *p*<0.001, *η_p_^2^* = 0.60, reflected that cycle time ratios were only affected by habitual speed in intentional imitation.

**Figure 6:**
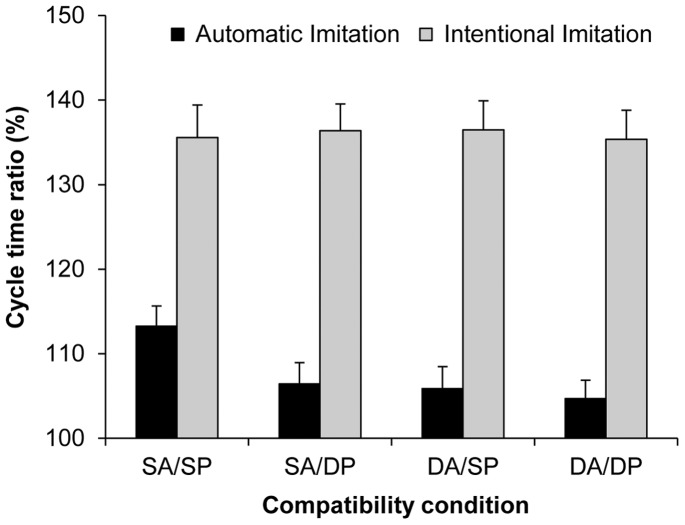
Automatic vs. intentional imitation: Cycle time ratios (%). Mean cycle time ratios (with standard error of the mean) for the factors intention, action type compatibility, and plane compatibility (SA = Same Action; DA = Different Action; SP = Same Plane; DP = Different Plane). The cycle time ratio in the distractor actions was 150%.

Importantly, the main effect of intention was significant, *F*(1, 18) = 517, *p*<0.001, *η_p_^2^* = 0.97, where cycle time ratios were closer to the display ratio for intentional compared to automatic imitation (136 vs. 108%, respectively; see Figure 6). Intention interacted significantly with action compatibility, *F*(1, 18) = 6.87, *p*<0.05, *η_p_^2^* = 0.28, as well as with plane compatibility, *F*(1, 18) = 5.87, *p*<0.05, *η_p_^2^* = 0.25. These results confirm that there were no differences across the four compatibility conditions for intentional imitation, and that in automatic imitation, the distractor ratio was more pronounced in the fully-compatible SA/SP condition over each of the incompatible condition. The three-way interaction involving intention, action type, and plane compatibility was only marginally significant, *F*(1, 18) = 3.44, *p* = 0.08, *η_p_^2^* = 0.16.

## Discussion

The present study contributes to a growing body of research demonstrating that observed kinematics automatically bias movement kinematics. First, our data show that observing a task-irrelevant rhythmical action during a brief motor preparation phase significantly biased the cycle time of subsequently executed rhythmical actions. However, this imitation bias was only a small fraction of the differences in the observed cycle times, as well as of the differences produced in intentional imitation. Second, the imitation bias was reduced, although still present, when the action type and/or plane differed across the observed and executed actions. Third, these effects were obtained at three separate time points: during execution immediately after offset of the distractor stimulus, and when there was a delay of 4 and 8 s between action observation and execution. Fourth, the effects were equally present when participants had no vision of their executing hand, as well as when online visual feedback was available. Before we discuss these main findings for automatic imitation in greater detail, we briefly turn to performance in intentional imitation, which served as a reference condition as it informed about possible deviations from the criterion movements, despite explicit instructions to copy them.

### Intentional Imitation Experiment

Subsequent to the main experiment, participants were asked to intentionally imitate the cycle times of the distractor movies with the same factorial manipulations as in the main study, except that only the 0 s delay condition was used. Overall, imitation performance was acceptably high. A few points are worth noting, however: For the habitually fast actions, participants slightly undershot the ratio of 150% between slow and fast ‘distractor’ speeds (140%), and more so for the habitually slow actions (132%). Such variance from the criterion range is not atypical for intentional imitation (e.g., [20], [23], [40]–[41]. Visual monitoring of the hand during execution tended to speed-up execution in intentional imitation, relative to performance with eyes closed, but this was at the cost of reduced cycle time ratios (Figure 5). Since these effects were relatively small and were not echoed in automatic imitation, we do not discuss them further. Importantly, with the exception of one just-significant effect that did not involve the distractor speed, cycle times were copied regardless of the compatibility between the imperative and distractor actions (Figure 6). This indicates that participants can easily extract the cycle time from an action in a different plane, or from an action that is different to the one they plan to execute, and can map it onto their own motor performance when instructed to do so.

### Automatic Imitation Experiment: Kinematic Fidelity and the Effects of Incompatible Distractor Stimuli

In the automatic imitation experiment we found robust effects of distractor speed for both habitually slow and habitually fast actions (Figure 3). These effects were markedly smaller than those in intentional imitation, despite identical stimulus conditions (modulation of 150%). In the compatible condition (SA/SP), the ratio of participants’ slow:fast cycle times in response to the different distractor speeds was 112%, and only 106% across the incompatible conditions (Figure 6). Expressed relative to intentional imitation, the modulation by the distractor speeds equated to 37% for the compatible condition, and to 16% for the incompatible conditions. Similarly, for discrete pointing movements, Bisio et al. [23] showed that reaching velocities in their implicit (compatible) imitation condition considerably undershot the modulations in the stimuli. A first general conclusion from this and our work is that modulations in distractor displays are typically not copied with high kinematic fidelity, in the sense of a 1∶1 match. Rather, participants’ kinematics normally only exhibit small, but reliable biases towards the distractor. Crucially, our study further demonstrates automatic imitation effects when the distractor action was different from the instructed action, as discussed next.

We correctly anticipated that the imitation bias would be attenuated in the three incompatible conditions, relative to the fully-compatible SA/SP condition. This result contrasts with intentional imitation, where participants copied the rhythms in compatible and incompatible conditions alike. The significant (though reduced) distractor effects in the incompatible conditions are particularly important in light of our analysis of the putative ‘distractor effects’ in earlier studies (e.g., [20], [23], see Introduction). In those studies, as well as in our compatible SA/SP condition, participants could have utilised the ‘task-irrelevant’ distractor as a valid guide for their own actions. Accordingly, we would submit that only the present incompatible conditions can be taken as evidence for genuine automatic imitation.

**Figure 7:**
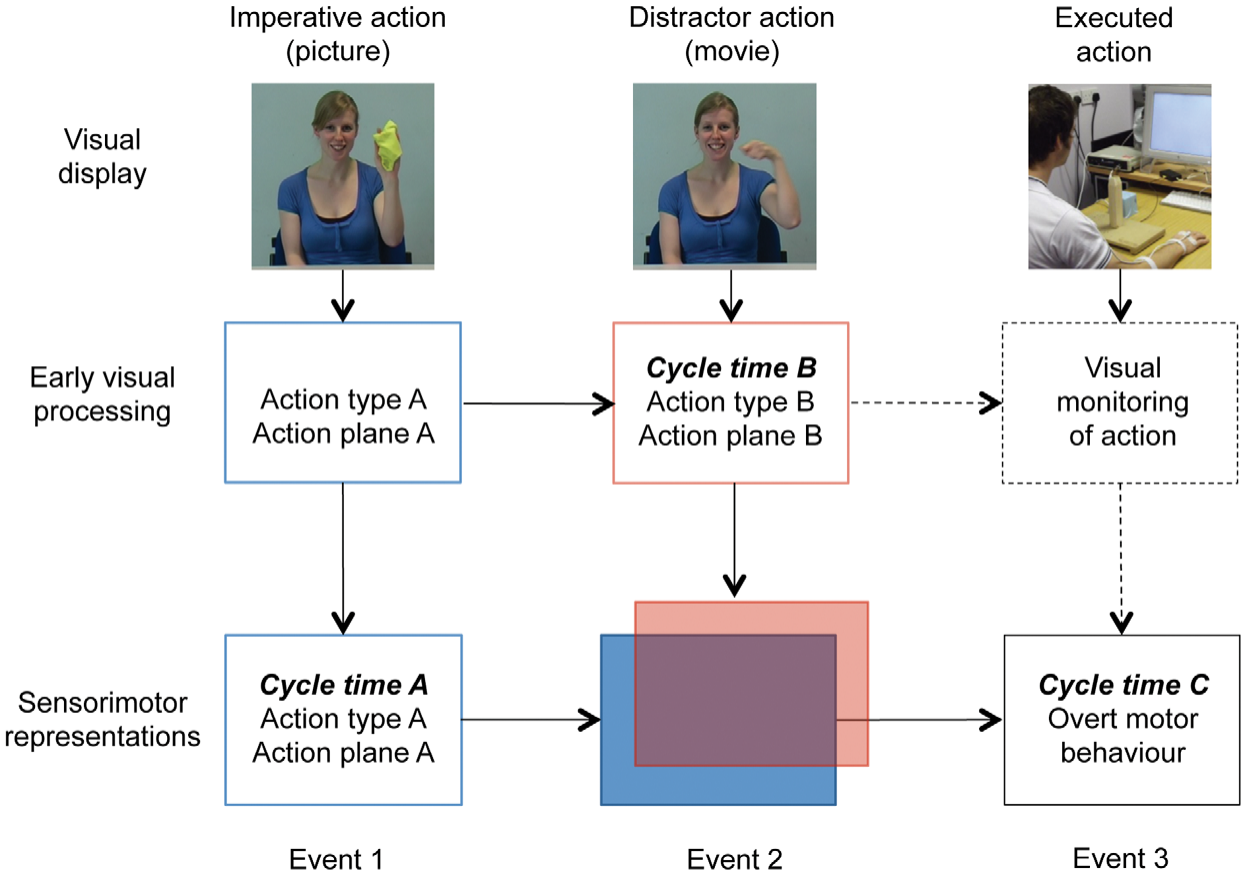
Hypothetical early-visual and sensorimotor processes during the three main events of an automatic imitation trial (for further details, see text). Event 1: Although the imperative picture did not specify execution speed, it is likely that the sensorimotor representation of the to-be-performed action included the habitual rhythm (cycle time A). Event 2: During presentation of the distractor movie, instructed and distractor actions are represented as two parallel and potentially competing sensorimotor streams. Event 3: The cycle time C during motor execution reflects the result of the biased competition for those two representations in Event 2. The dotted route indicates an alternative explanation of the imitation bias, via visual monitoring and related corrections during execution, for which no evidence was found in the present study.

Why were distractor effects reduced (while still statistically significant) in the present incompatible conditions, relative to the compatible SA/SP condition? We first provide an account based on action and plane compatibility as separable factors, and then propose a more integrative account based on competing sensorimotor representations. Given the crucial role of action goals in action observation and imitation, it is plausible that a distractor action with the same goal as the imperative action is not only more likely to be imitated as an *action category* (as demonstrated in RT studies on automatic imitation; [3]), compared to a DA/SP distractor, but also that low-level distractor features, such as its rhythm, are imitated more accurately. A similar case could be made for our plane compatibility manipulation, as sensorimotor synchronisation improves with compatible spatial information [36], [42]. Although the present offline paradigm did not involve overt synchronisation, it is likely that participants covertly synchronised the observed distractor action with their own motor planning, as described next. Figure 7 shows the three main events of the present paradigm, along with hypothetical visual and sensorimotor processes. Although the imperative picture stimulus *per se* did not specify a particular rhythm, participants had been instructed to perform the habitually slow and fast actions at different speeds. This was reinforced throughout the experiment by the fact that, for example, habitually slow imperative actions were always followed by a distractor movie of a habitually slow action (performed at 60 or 90 bpm). We therefore assume that, upon seeing the imperative stimulus (Event 1), participants formed a sensorimotor representation which included that action’s habitual rhythm (slow or fast). During presentation of the distractor movie (Event 2), participants were then able to simulate the instructed action in real-time [43], and to synchronise this internal simulation with the observed distractor action (overlapping boxes in Figure 7). According to Hove et al. [36], for example, a tighter synchronisation would then be predicted for spatially compatible actions. As a result, during motor execution (Event 3), cycle time C would be more strongly biased by distractor actions with matching, compared to non-matching, planes.

Whereas the above account of reduced distractor effects in the incompatible conditions is well in line with the existing literature (see Introduction), two of our present findings do not fit. First, this account should in principle also apply to intentional imitation, but we found no related differences between compatibility conditions in intentional imitation. In addition, we found significant interactions between intentionality of imitation and each of the compatibility factors: results which underline that the attenuation by incompatible distractor actions was specific for automatic imitation. Second, the effects of action and of plane compatibility in automatic imitation were not additive. That is, no further attenuation was observed when both action and plane were incompatible (DA/DP), relative to the other two incompatible conditions. If, however, one assumes separable contributions of action and plane compatibility, the DA/DP condition should show the strongest attenuation, and there should be no interaction between action and plane compatibility - neither of which was the case in the present study. We are therefore inclined to favour a more integrative account, according to which the distractor’s impact on motor processing is generally reduced whenever this is not functional for the observer’s own motor planning, as developed further in the following.

It is useful to conceptualise automatic imitation effects, such as in the present paradigm, in the framework of Cisek and Kalaska’s [44] biased competition hypothesis. During Event 2 (distractor presentation), the instructed and distractor actions can be modelled as two parallel, and potentially competing sensorimotor streams. In case that the distractor action is identified as action-irrelevant (all incompatible conditions), the competition between these two streams is strongly biased towards the instructed action. Consequently, attempts to covertly synchronise the two streams will be relatively sparse. Motor preparation then proceeds largely (but not necessarily completely) decoupled from the available visual input, which would result in relatively small distractor effects during subsequent execution, as observed in the present study. In contrast, when the distractor action widely overlaps with the intended action (compatible SA/SP condition), the two corresponding sensorimotor streams merge, rather than compete. Covert synchronisation between intended and observed actions will be enhanced relative to the incompatible conditions, and this will, in turn, result in stronger distractor effects during execution. In summary, in this biased competition account, the strength of distractor effects depends on the general usefulness of the concurrent visual input for supporting motor preparation, whereas our earlier account assumed separate effects of matching goals and planes. Further research will be required to identify the conditions under which the imitation bias might (a) approach those in intentional imitation (e.g., by manipulating the ratio of compatible and incompatible trials, or the action-relevance of incompatible distractor trials), and (b) might be further reduced or even annihilated. In addition, the proposed role of covert synchronisation between distractor action and simulation of the to-be-executed action, as a core mechanism of the observed imitation bias, also requires further investigation.

### Automatic Imitation Experiment: Effects of Delay and Visual Monitoring

The third aim of the present study was to assess if the imitation bias would reduce when a short delay is inserted between Events 2 and 3, that is, between the end of the distractor presentation and movement onset. In previous RT studies, automatic imitation effects have been shown to decay after delays shorter than 1 s [7], [45]–[46]. In reach trajectory, a similarly rapid decay was found after the self-priming from one’s own earlier reach path [47]. In contrast, Edwards et al. [30] demonstrated an imitation bias in reach-grasp kinematics following a longer (3 s) delay between action observation and execution. Relative to the generally short-lived priming effects in the above studies, we found a remarkable stability of the distractor effects across the three delay conditions (0, 4 and 8 s, see Figure 4). The only exception was a pocket of slightly enhanced distractor effects for immediate execution in a small subset of the data, namely fully-compatible, habitually slow actions. Whilst this finding requires further study (and fits well to the above framework), the dominant explanandum in the present results is certainly the relative longevity of the imitation bias compared to previous results. Since participants were not prevented from covert rehearsal of the instructed action in the delay period, the most likely explanation is that they continued to simulate this action, including its rhythm, in the delay period up until execution. In support of this interpretation, Vogt [41] documented visual and motor imagery as effective rehearsal strategies for maintenance of temporal information in short-term memory over 12 s. Thus, one likely factor distinguishing the present study from studies showing rapid decay of automatic imitation is the opportunity for covert rehearsal. Further factors could be the relatively long presentation duration of the distractor in the present study (4 s), as well as the nature of the dependent variable, given that Vogt et al. [7] and Gowen et al. [45] used reaction times as dependent measures, whereas the present study employed cycle times.

The fourth aim of this study was to assess possible effects of visual monitoring of one’s own hand during execution, primarily in automatic imitation. If the imitation bias had been present only when vision of the hand was available during execution, then an explanation in terms of a purely visual (rather than visuo*motor*) representation of the distractor action could have accounted for the findings. Exploring performance without vision of the effector (see [21], [24]) is particularly relevant in a relatively slow experimental paradigm such as ours, which provides the opportunity for vision-based corrections during execution. In contrast to intentional imitation, where visual monitoring indeed affected cycle time ratios (see Discussion above), automatic imitation effects were equally pronounced in the vision and no-vision groups (Figure 3). Importantly, this finding allows us to rule out a ‘visual’ interpretation of the observed imitation bias, according to which a purely visual representation of the distractor would subsequently be compared to the visual feedback available during motor execution, and used for related online corrections (dashed lines in Figure 7; note, however, that these results do not rule out corrections based on kinesthetic input during execution [48]). By way of exclusion then, the present finding confirms that the observed distractor effects are primarily due to visuo*motor* interactions, as well as biased competition, during observation of the distractor actions.

Finally, it is tempting to speculate about the possible neural substrate of the observed imitation bias in rhythmical actions. One piece of evidence comes from an unpublished pilot study on the imitation of rhythmical actions in a patient with visual form agnosia [49]. Despite the patient’s severe impairment of ventral visual stream function, D.F. [50] showed a remarkable accuracy in immediate unintended imitation of the (manipulated) cycle times in a variety of everyday actions (r = 0.90 between displayed and produced cycle times in the pilot study). It is thus conceivable that, in addition to information about action-relevant object properties, information about the temporal characteristics of an observed action is also initially processed via the dorsal visual stream (see also [51]). This does, however, not exclude that additional brain structures become engaged during covert simulation. Likely candidates involved in the observation and execution of rhythmical actions are Broca’s area, the basal ganglia, and the cerebellum [52]–[53].

### Conclusions

In the present study we used everyday rhythmical actions to explore a core dimension of imitative alignment or ‘low-level motor resonance’ [8]. The main finding was a reliable bias in response cycle times as a result of the (task-irrelevant) modulation in distractor cycle times. This imitation bias was robust against manipulations of the time interval before motor execution and of visual monitoring of the hand during execution, where the latter indicates that this is a genuine visuomotor effect. Importantly, the imitation bias was still present, though attenuated, in the incompatible conditions where the distractor action was not useful for motor preparation. We submitted that these incompatible conditions provide a more cogent demonstration of automatic imitation than compatible conditions (as used, for e.g., by [20], [23]), where distractor displays can principally be used to support motor preparation. That is, the distractor effects in the present incompatible conditions occurred even in a situation where participants had good reasons to minimise the impact of the observed distractor actions on their own motor preparation, in order to avoid confusion between the conflicting actions. Given that the effects of action and of plane compatibility were not additive, and that they were not found in intentional imitation, we favoured an account in which the distractor’s impact on motor processing can be generally reduced over an account where action and plane incompatibility reduce the imitation bias independently. Using Cisek & Kalaska’s [44] framework, we further conceptualised the suppression in incompatible trials as the result of a biased competition between the sensorimotor representations of distractor and instructed action. The notion of a global suppression effect, where potentially all parameters of the observed action are affected, was principally supported by the fact that, although the conflict between distractor and instructed actions was only applied to action type and plane, the compatibility manipulations clearly affected an additional parameter, namely the cycle times.

As such, our study is the first of its kind involving multiple compatibility dimensions and studying their interdependency. The results indicate a rather moderate kinematic fidelity between distractor and executed action, not unlike the modest modulations of reaction times in conventional automatic imitation paradigms [3]. Nevertheless, we hope that the present study paves the way for further investigations into competitive processes between quasi-encapsulated sensorimotor representations, as well as into the boundary conditions for both near-perfect imitative behaviour and the more moderate imitative biases found when strategic factors are carefully controlled.
